# Soft tissue coverage on the segmentation accuracy of the 3D surface-rendered model from cone-beam CT

**DOI:** 10.1007/s00784-016-1844-x

**Published:** 2016-05-21

**Authors:** J. K. Dusseldorp, H. C. Stamatakis, Y. Ren

**Affiliations:** Department of Orthodontics, University of Groningen, University Medical Centre Groningen, Hanzeplein 1, UMCG, 9700 RB Groningen, The Netherlands

**Keywords:** Cone-beam computed tomography, Segmentation, Accuracy, Surface models, Soft tissues

## Abstract

**Objectives:**

The aim of this study is to investigate the effect of soft tissue presence on the segmentation accuracy of the 3D hard tissue models from cone-beam computed tomography (CBCT).

**Materials and methods:**

Seven pairs of CBCT Digital Imaging and Communication in Medicine (DICOM) datasets, containing data of human cadaver heads and their respective dry skulls, were used. The effect of the soft tissue presence on the accuracy of the segmented models was evaluated by performing linear and angular measurements and by superimposition and color mapping of the surface discrepancies after splitting the mandible and maxillo-facial complex in the midsagittal plane.

**Results:**

The linear and angular measurements showed significant differences for the more posterior transversal measurements on the mandible (*p* < 0.01). By splitting and superimposing the maxillo-facial complex, the mean root-mean-square error (RMSE) as a measurement of inaccuracy decreased insignificantly from 0.936 to 0.922 mm (*p* > 0.05). The RMSE value for the mandible, however, significantly decreased from 1.240 to 0.981 mm after splitting (*p* < 0.01).

**Conclusions:**

The soft tissue presence seems to affect the accuracy of the 3D hard tissue model obtained from a cone-beam CT, below a generally accepted level of clinical significance of 1 mm. However, this level of accuracy may not meet the requirement for applications where high precision is paramount.

**Clinical relevance:**

Accuracy of CBCT-based 3D surface-rendered models, especially of the hard tissues, are crucial in several dental and medical applications, such as implant planning and virtual surgical planning on patients undergoing orthognathic and navigational surgeries. When used in applications where high precision is paramount, the effect of soft tissue presence should be taken into consideration during the segmentation process.

## Introduction

Low-dose cone-beam computed tomography (CBCT) for three-dimensional (3D) imaging of the maxillo-facial structures is increasingly used in the latest years in the medical and dental field, and it extends to a wide range of applications [1–3]. Accurate visualization of the face, jaws, and its components are important for clinical diagnostics and decision-making [4]. Nowadays, the application of 3D cephalometric analysis plays an important role in cases of complex maxillo-facial abnormalities [5, 6] and for the evaluation of growth or treatment outcomes [7–9]. Accurate 3D surface-rendered models, especially of the hard tissues, are crucial in applications such as virtual surgical planning on patients undergoing orthognathic surgery, during dental implant and prosthetic procedures, and when simulating treatment outcomes [10–15].

Surface-rendered 3D models of the hard tissues derived from the CBCT Digital Imaging and Communication in Medicine (DICOM) data that are originally composed of voxels, each with its own gray value based on the radiation absorbed by the tissues during the scan. By means of specific software applications, reconstructions are possible in sagittal, coronal, and axial planes, allowing the operator to scroll through the tissue structures in any direction of interest. Most applications also include reconstruction techniques allowing image extraction out of the DICOM data similar to those from conventional radiographic techniques, such as orthopantomograms and lateral cephalometric radiographs [16]. In order to construct a 3D digital model of specific tissues out of the original voxel-based data, a specific process called segmentation has to be followed. Briefly, the operator provides the software an upper and a lower threshold matching the gray-level range of the voxels which specifies the tissues of interest. The software then discards all data outside these limits and depicts only the voxels within the set threshold values.

Factors influencing the quality and accuracy of the models provided by the segmentation process can be divided in three main categories [17]. The first comprises CBCT system factors, such as scanner type, field of view settings, and voxel size settings [18–21]. The second category is patient-related factors such as positioning of the patient in the scanner, metal artifacts, and the soft tissue covering [22–25]. The third are operator-related factors, such as the segmentation process itself, the employed software, and the operator performing the segmentation [26–29]. Most studies published on this particular subject were based on research using dry skulls.

The use of the dry skull without any soft tissue substitute may present a drawback, as it does not simulate the human anatomy in real life. The soft tissues are sources of scattering radiation during a radiographic examination, resulting in deterioration of the signal-to-noise ratio and, subsequently, of the gray-level value differentiation in voxels between different density tissues, presumably resulting to lower bone image quality [30]. As a result, measurements obtained from images on a human dry skull may deviate from the “true” values if they be obtained from the same subjects with soft tissue coverage. Interpretation of these results may be misleading for decision-making in clinical practice. To overcome this error, some studies introduced latex balloons filled with water as a soft tissue equivalent [31, 32]. One of the drawbacks of this method is that it does not reflect the soft tissue properties or distributions in real life. Alternatively, human cadavers may represent better the human anatomy, which are, however, difficult to obtain. The effect of soft tissue presence on the segmentation process and the resulting 3D surface-rendered volumetric models is not yet well documented, specifically when all the above-mentioned affecting side parameters are standardized.

Therefore, the aim of this study is to investigate whether the soft tissue presence affects the segmentation accuracy of the 3D surface-rendered volumetric models from cone-beam CT in a setting in which other affecting parameters were controlled.

## Materials and methods

### CBCT Data acquisition

A sample of seven DICOM datasets was used for this study, containing the scans of seven anonymous cadaver heads comprising both edentulous and partially edentulous jaws that were initially scanned with the KaVo 3D exam scanner (KaVo Dental GmbH, Bismarckring, Germany) using a standardized scanning protocol [28]. Subsequently, the cadavers were meticulously macerated according to an established protocol by the Department of Anatomy, University Medical Centre Groningen, Groningen, The Netherlands [33]. This procedure resulted in the respective seven dry skulls that were scanned with the same scanner and following the original protocol as used during the cadaver scans. The dry skulls were repositioned in the scanner using the laser reference lines of the unit [34]. Yaw, pitch, roll, and height settings were obtained from the first series of cadaver scans by means of the manufacturer’s software in order to achieve similar positioning. For both sets of scans, a 0.3-mm voxel size with a 17-cm field of view was used. Therewith the complete sample used in this study consists of DICOM datasets of two groups, scans of the cadaver heads with soft tissue, and scans of the respective dry skulls without soft tissue.

### The segmentation protocol

The DICOM datasets were exported from the unit’s dedicated software and imported into ITK-SNAP, a specialized segmentation software package for medical imaging [35]. The procedure starts with determining the region of interest and threshold levels, followed by setting the seeding points in the tissues of interest, which results in a very close approximation of the 3D structures with neighboring intensities. The final 3D surface-rendered model of the bone surface is formed by segmenting using the level-set method to drive the active contour evolution as coded in the ITK-SNAP software [35]. It is based on region competition causing the active contour to reach equilibrium at the boundary of the regions, i.e., the borders of the hard tissues, subsequently discarding pixels representing the surrounding soft tissues. The segmentation procedure for the DICOM datasets was performed in a random order following a segmentation protocol fitting the software package. This procedure is partly operator dependent, since each cadaver and dry skull requires an individual set of segmenting thresholds. All segmentations were performed by one operator and repeatedly three times with a 1-week interval to determine intraobserver reliability. This segmentation procedure resulted in digital 3D surface-rendered hard tissue models for both groups, with and without the presence of soft tissues.

### Measuring procedure

**Fig. 1:**
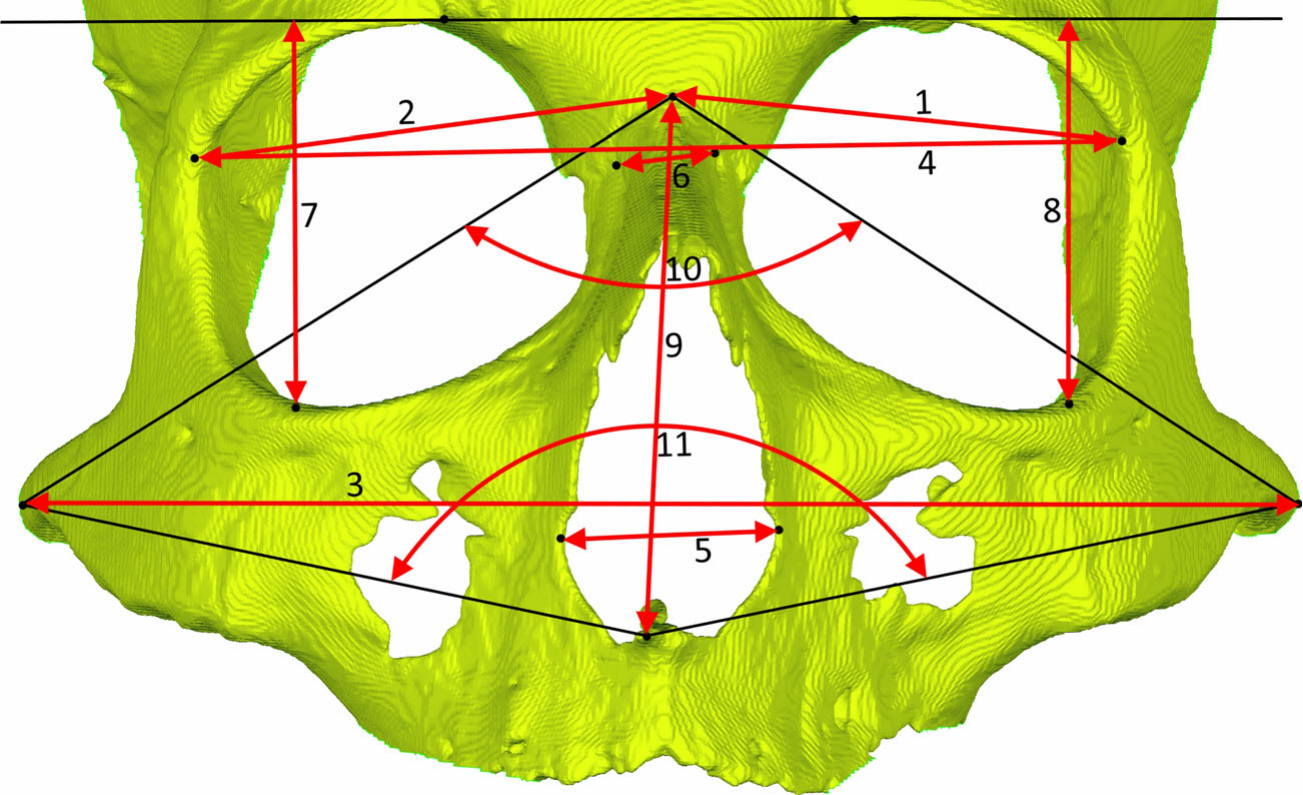
Linear and angular measurements on the maxillo-facial complex. *1*, *2* orbita width L (left) and R (right)—distance between points medio-orbitale and zygomaticofrontal medial suture; *3* zygion width—distance between points zygion R and L; *4* frontozygomatic width—distance between points zygomaticofrontal medial suture R and L; *5* nasal canal width—distance between points lateral piriform aperture R and L; *6* nasal width—distance between points medio-orbitale R and L; *7*, *8* orbita height—distance between point orbitale and the line crossing both supraorbital points; *9* nasion–anterior nasal spine height—distance between points nasion and ANS; *10* facial divergence angle nasion—angle from zygion R point to nasion point to zygion L point; *11* facial divergence angle ANS—angle from zygion R point to ANS point to zygion L point

**Fig. 2:**
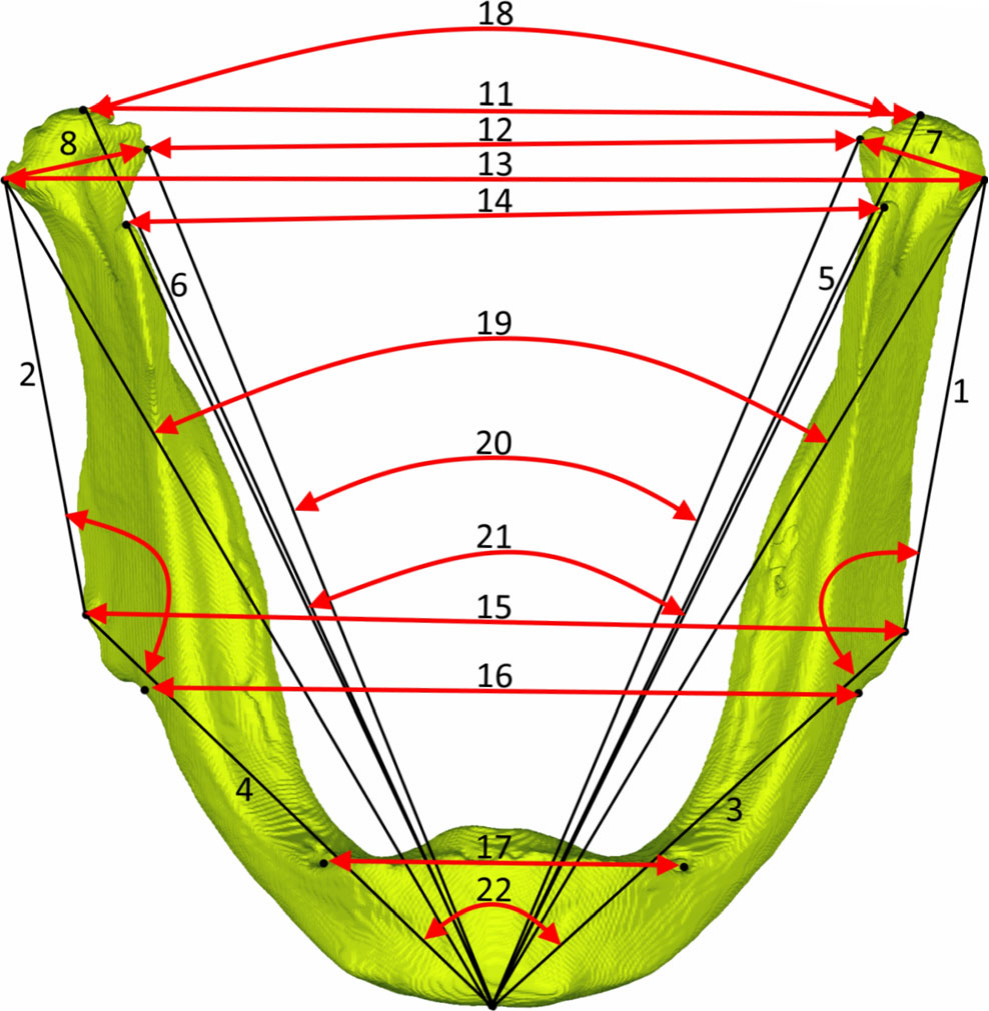
Linear and angular measurements on the mandible. *1*, *2* ramus length (Co–Go); *3*, *4* mandibular body length (Me–Go); *5*, *6* total mandibular length (Co–Me); *7*, *8* condyle width (CoLat–CoMed); *9*, *10* gonial angle (Co–Go–Me); *11* mandibular width at condylion (Co R–Co L); *12* mandibular width at condylion laterale (CoLat R–CoLat L); *13* mandibular width at condylion mediale (CoMed R–CoMed L); *14* mandibular width at coronoid process (CP R–CP L); *15* mandibular width at gonion (Go R–Go L); *16* mandibular width at antegonion (AG R–AG L); *17* mandibular width at mentale foramina (Men.for R–Menfor L); *18* condylion divergence angle (Co R–Me–Co L); *19* condylion laterale divergence angle (CoLat R–Me–CoLat L); *20* condyle mediale divergence angle (CoMed R–Me–CoMed L); *21* coronoid divergence angle (CP R–Me–CP L); *22* gonion divergence angle (Go R–Me–Go L); *23* antegonion divergence angle (AG R–Me–AG L)

The effect of soft tissue on the 3D surface-rendered hard tissue models was analyzed by comparing the two groups of models. The models representing the dry skulls were set as the reference 3D models, against which the models deriving from the cadavers with the soft tissues were tested. Two different methods were employed for comparisons. The first method was based on linear and angular measurements using the Simplant O&O software (Materialise Dental, Leuven, Belgium). A series of landmarks were selected and identified on the 3D digital models, and subsequently, the defined linear and angular measurements were performed by the software [32, 33, 36]. These measurements are illustrated in Figs. 1 and 2 for the maxillo-facial complex and the mandible, respectively, and were assessed three-dimensionally on the surface-rendered models. The landmark identifications were performed blindly and in random order by one operator, three times repeatedly with a 1-week interval to test for intraobserver reliability. The second method was based on volume superimposition and color mapping of the surface differences, which are described in the following session in detail [37, 38].

### Superimposition and color mapping

This procedure was performed using Geomagic Studio software package (Geomagic Solutions, USA). First, the two regions of interest were defined, namely the maxillo-facial complex and the mandible. In this study, the maxillo-facial complex was defined as the area including all external facial surfaces below a horizontal plane, passing through the highest point of the orbital rims, parallel to the Frankfurt horizontal, and ventral to a vertical coronal plane which crosses both temporo-zygomatic sutures, excluding the mandible. The second region of interest was the mandible which was compared completely, including all external lingual and facial surfaces.

**Fig. 3:**
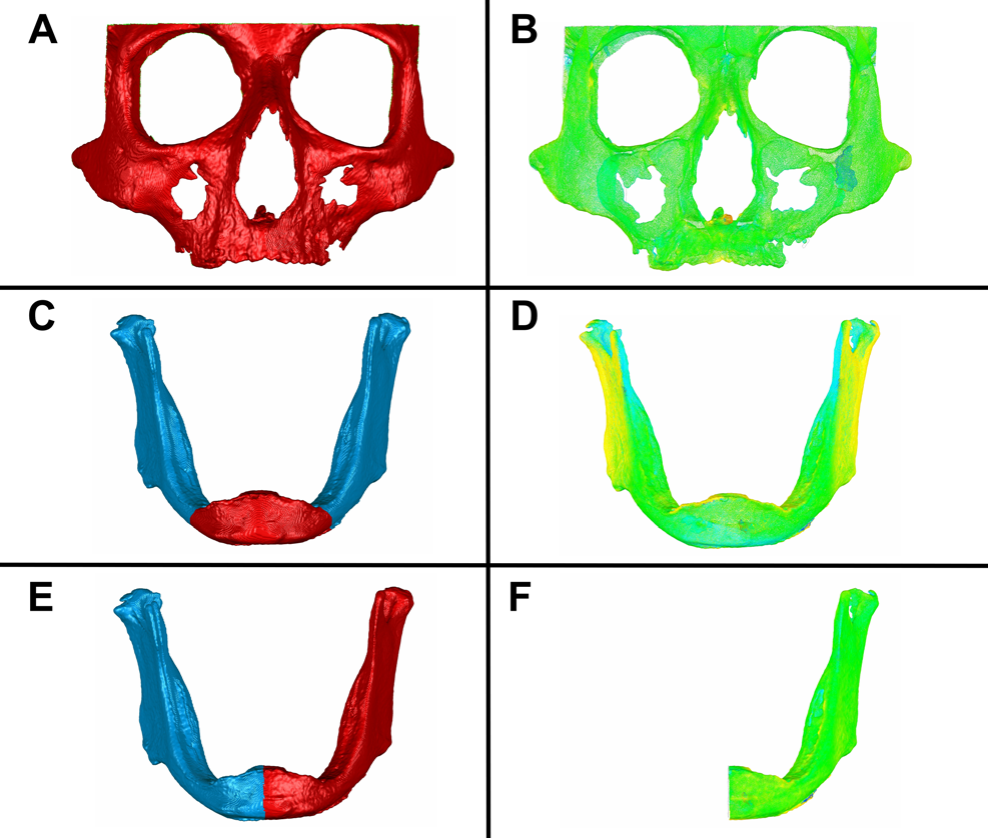
Registration and volume comparisons of the maxillo-facial complex and the mandible. **a**Selection of the defined maxillo-facial complex and automatic registration by the best-match fitting method. **b** Volume comparison of the maxillo-facial complex. **c** Selection of the defined frontal area of the mandible and automatic registration by the best-match fitting method. **d** Volume comparison of the complete mandible. **e** Selection of the left mandibular side and automatic registration by the best-match fitting method. **f** Volume comparison of the left mandibular side

Before comparing, the models had to be superimposed (Fig. 3). For both regions of interest, an automatic operator-independent matching was followed; the software performs the best-fit matching based on the least squared differences between the models for optimal superimposition. The maxillo-facial complexes were matched on their complete external facial surfaces. The mandibles, however, given the known potential transversal dimensional deformation due to the maceration and drying techniques [39], were matched on their frontal area defined as all external surfaces of the mandible ventral to a vertical coronal plane passing through both mental foramina (Fig. 3c). In addition, both the maxillo-facial complex and the mandible models were split in their midsagittal planes in order to further minimize any interfering effect of such dimensional deformation and isolate the soft tissue effects. The resulting left and right parts were matched and compared separately (Fig. 3e, f).

The differences between the models were displayed by means of color mapping of surface deviations and by sampling the models to approximately one million polygons each. In addition, the software provides information for each comparison indicating the magnitude of deviation between the surfaces of the two registered volumes. The signed average of the surface differences presents the absolute mean of the surface differences by simply adding up positive and/or negative distances for all surface polygons. By definition, differences are marked positive when the tested surface lies outside the reference surface and vice versa. The root-mean-square error (RMSE) was used as an absolute measure of model surface deviations, in order to account for positive and negative differences which otherwise could cancel out each other [38, 40]. In addition, the mean values of the left and right sides were calculated.

### Statistical analysis

The reliability of all linear and angular measurements was expressed by intraclass correlation coefficients (ICC) for absolute agreement based on a two-way random effects analysis of variance (ANOVA) between the three repeated measurements. All variables were positively tested for normality using the Shapiro-Wilk test with all *p* values being >0.05. The significance of differences between the measurements performed on both groups and the differences between RMSE values was calculated using the paired sample *T* test. The level of clinical significance was set at 0.05. The RMSEs and signed average of differences were calculated for the selected surfaces on each maxillo-facial complex and mandible model. Mean and standard deviations were calculated. All statistical analyses were performed using Statistical Package of Social Sciences (SPSS).

## Results

### Measurement reliability

**Fig. 4:**
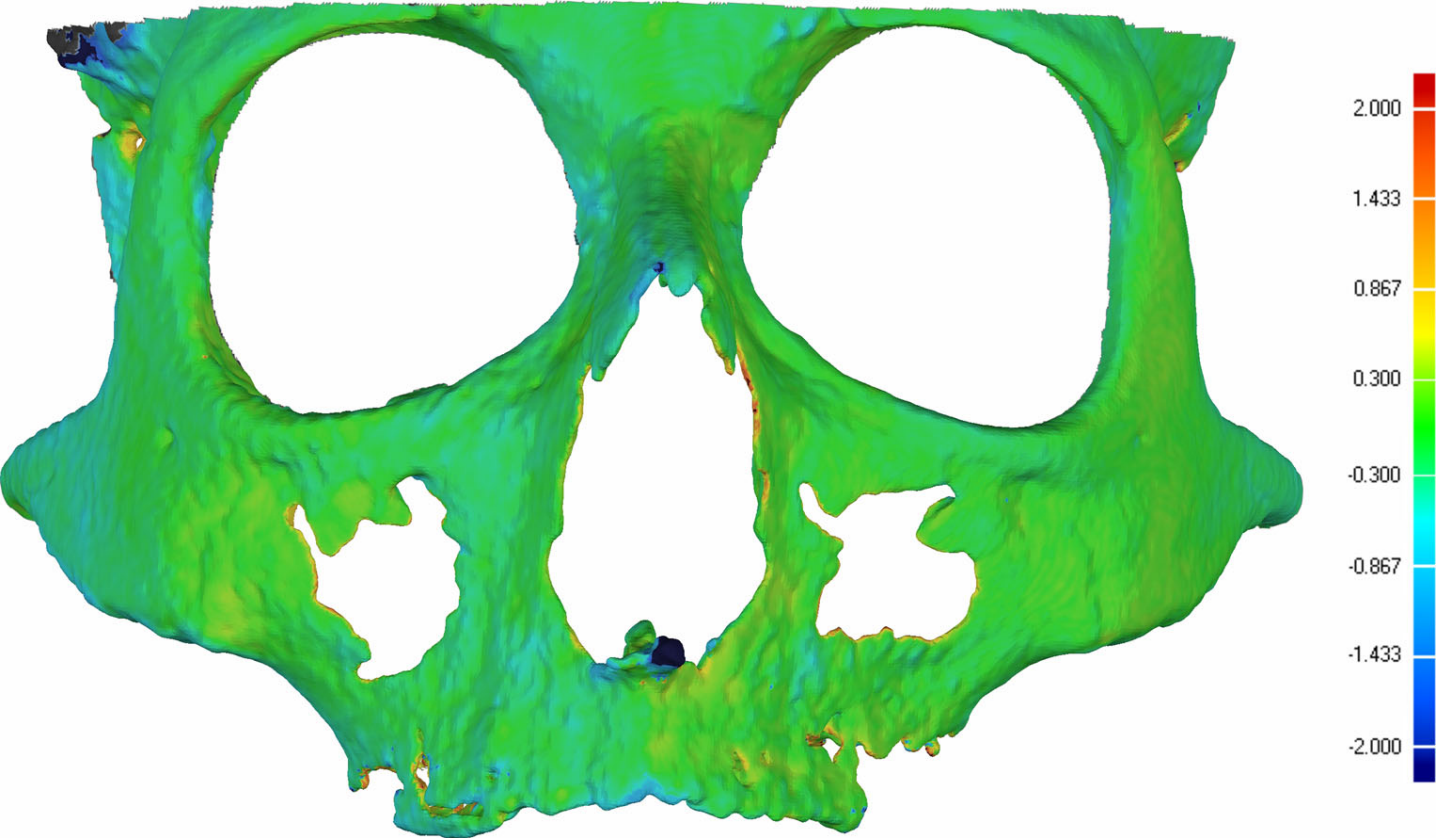
An illustration of the segmentation reliability based on color mapping. The segmentation reliability was assessed by superimposing and color mapping two surface models of the same maxillo-facial complex segmented at two different sessions. *Green color* represents differences within 0.3 mm which equals the voxel size

No differences between the repeated segmentations were found exceeding the level of 0.3 mm, which was the voxel size (Fig. 4). These values confirm the reliability of the used segmentation protocol as being excellent. The ICCs of the reliability tests on all measurements obtained from both reference and test groups varied within 0.94–1.00 and were accordingly classified as being excellent.

### Linear and angular measurements on the maxillo-facial complex

The results from the linear measurements performed on the mandible were shown in Table 1. The mean differences for all linear measurements on the maxillo-facial complex ranged between −0.41 and −0.78 mm (Table 1). The differences between the reference and test groups for the angular measurements were between −0.41° and −0.12°. Both were found statistically non-significant (*p* > 0.05).

**Table 1:** Results of the linear and angular measurements on the maxillo-facial complex

#	Measurement	Unit	Group with soft tissue		Group without soft tissue		Comparison	
			Mean	SD	Mean	SD	Δ	95 % CI
	Linear							
1	Orbita width L	mm	44.76	2.84	44.87	2.82	−0.25	−1.83–1.31
2	Orbita width R	mm	45.72	2.54	45.59	2.87	0.08	−0.76–0.91
3	Zygion width	mm	131.38	6.95	131.28	6.93	0.10	−0.59–0.78
4	Frontozygomatic width	mm	98.01	5.65	97.58	5.67	0.11	−0.08–0.32
5	Nasal canal width	mm	25.19	2.77	24.44	2.40	0.74	−0.34–1.82
6	Nasal width	mm	12.18	2.69	11.80	2.43	0.38	−0.99–1.75
7	Orbita height R	mm	38.83	1.62	38.66	1.54	0.17	−1.25–1.58
8	Orbita height L	mm	38.79	1.91	38.00	2.16	0.78	−0.48–2.04
9	Nasion–anterior nasal spine height	mm	53.95	2.93	54.32	3.12	−0.36	−0.77–0.04
	Angular							
10	Facial divergence angle nasion	°	88.49	3.65	88.61	3.27	−0.12	−1.99–1.76
11	Facial divergence angle ANS	°	86.72	2.57	87.13	2.13	−0.41	−2.18–1.35

Mean values. Standard deviations (SD). Average differences (Δ) with confidence intervals (CI) at 95 % and level of significance of the differences (P)

### Linear and angular measurements on the mandible

The results from the linear measurements performed on the mandible were shown in Table 2. Significant differences were found for the mandibular width at the coronoid process (1.95 mm, *p* < 0.01), at condylion laterale (1.42 mm, *p* < 0.01) and at condylion medial (3.60 mm, *p* < 0.001). The angular measurements showed significant differences for the coronoid divergence angle (1.67°, *p* < 0.001), the condyle lateral divergence angle (1.22°, *p* < 0.01), and the condyle medial divergence angle (1.85°, *p* < 0.001).

**Table 2:** Results of the linear and angular measurements on the mandible

#	Measurement	Unit	Group with soft tissue		Group without soft tissue		Comparison	
			Mean	SD	Mean	SD	Δ	95 % CI
	Unilateral							
	Linear							
1	Ramus length L	mm	58.50	4.79	59.95	4.28	−1.45	−3.27–0.37
2	Ramus length R	mm	59.43	5.44	59.69	4.01	−0.26	−2.19–1.68
3	Mandibular body length L	mm	86.80	6.35	86.62	5.54	0.18	−1.23–1.58
4	Mandibular body length R	mm	87.50	4.89	87.50	5.24	−0.00	−1.22–1.22
5	Total mandibular length L	mm	124.01	7.03	125.49	7.35	−1.48	−2.80–−0.15
6	Total mandibular length R	mm	124.47	6.88	124.62	6.96	−0.15	−2.68–2.38
7	Condyle width L	mm	20.05	2.38	20.68	2.93	−0.63	−1.70–0.43
8	Condyle width R	mm	20.88	2.50	21.88	2.59	−1.00	−2.31–0.31
	Angular							
9	Gonial angle L	°	115.83	3.65	116.63	4.02	−0.80	−1.88–0.29
10	Gonial angle R	°	114.40	4.30	114.38	3.50	0.02	−2.10–2.14
	Bilateral							
	Linear							
11	Mandibular width at condylion	mm	103.73	9.10	103.32	6.42	0.41	−3.58–4.41
12	Mandibular width at condylion laterale	mm	123.36	6.18	121.95	5.63	1.42**	0.60–2.23
13	Mandibular width at condylion mediale	mm	85.87	3.44	82.28	2.60	3.60***	2.70–4.50
14	Mandibular width at coronoid process	mm	98.20	7.64	96.24	7.00	1.95**	1.16–2.74
15	Mandibular width at gonion	mm	97.41	5.99	96.29	5.64	1.12	−0.29–2.54
16	Mandibular width at antegonion	mm	84.75	5.38	83.90	4.57	0.86	−1.28–2.99
17	Mandibular width at mentale foramina	mm	47.14	4.91	46.06	3.04	1.08	−1.72–3.89
	Angular							
18	Condylion divergence angle	°	49.44	4.53	48.85	2.68	0.59	−1.55–2.73
19	Condyle laterale divergence angle	°	60.80	2.07	59.58	1.66	1.22**	0.64–1.79
20	Condyle mediale divergence angle	°	41.95	1.70	40.10	1.82	1.85***	1.32–2.38
21	Coronoid divergence angle	°	55.59	2.44	53.92	2.11	1.67***	1.15–2.19
22	Gonion divergence angle	°	68.10	4.02	67.29	4.32	0.80	−0.55–2.15
23	Antegonion divergence angle	°	78.90	3.69	78.32	3.79	0.58	−0.21–1.36

Mean values. Standard deviations (SD). Average differences (Δ) with confidence intervals (CI) at 95 % and level of significance of the differences (P)

*p < 0.05, **p < 0.01, ***p < 0.001

### Volume comparisons of the maxillo-facial complex

The results of the volume superimposition and surface comparisons of the maxillo-facial complex between the reference and test models were shown in Table 3. The mean RMSE was 0.936 mm with a standard deviation of 0.188 mm comparing the complete maxillo-facial complexes. The mean signed average of difference between surfaces was −0.092 mm with all values being negative. Dividing the maxillo-facial complex to a left and right side for separate registration and comparing procedures decreased the mean RMSE to 0.922 mm with a standard deviation of 0.195 mm which was not significant (*p* > 0.05).

**Table 3:** Results of the volume comparisons of the maxillo-facial complex

	Registration: best fit on the complete external facial surfaces of the maxillo-facial complexes		Registration: best fit on the concerning left or right side	
	Signed average of difference between surfaces (mm)	RMSE of difference between surfaces (mm)	Signed average of differences between surfaces (mm)	Mean RMSE values of differences between surfaces (mm)
1	−0.058	0.582	−0.045	0.539
2	−0.078	1.084	−0.077	1.071
3	−0.021	1.064	−0.020	1.043
4	−0.197	1.089	−0.197	1.075
5	−0.027	0.886	−0.018	0.872
6	−0.103	0.817	−0.119	0.828
7	−0.162	1.030	−0.167	1.026
Mean	−0.092	0.936	−0.092	0.922
SD	0.066	0.188	0.071	0.195

Surface differences indicating inaccuracy of 3D digital segmented models, derived from CBCT, caused by the presence of soft tissue

### Volume comparisons of the mandible

The results of the mandible comparisons were shown in Table 4. Analyzing the complete mandibles registered on the frontal area resulted in a mean RMSE of 1.240 mm with a standard deviation of 0.375 mm. The mean signed average of difference between surfaces was −0.189 mm with all values being negative. Dividing the mandible in a left and right side, for separate registration and comparing procedures, significantly decreased the mean RMSE to 0.981 mm with a standard deviation of 0.411 mm (*p* < 0.01).

**Table 4:** Results of the volume comparisons of the mandible

	Registration: best fit on symphysis surfaces		Registration: best fit on the concerning left or right side	
	Signed average of difference between surfaces (mm)	RMSE of difference between surfaces (mm)	Signed average of differences between surfaces (mm)	Mean RMSE values of differences between surfaces (mm)
1	−0.077	0.879	−0.052	0.503
2	−0.221	1.422	−0.214	1.073
3	−0.290	1.886	−0.273	1.640
4	−0.164	0.956	−0.123	0.798
5	−0.181	1.118	−0.121	0.694
6	−0.056	0.921	−0.032	0.758
7	−0.333	1.496	−0.271	1.401
Mean	−0.189	1.240	−0.155	0.981
SD	0.102	0.375	0.099	0.411

Surface differences indicating inaccuracy of 3D digital segmented models, derived from CBCT, caused by the presence of soft tissue

## Discussion

The aim of this study was to investigate whether the soft tissue covering affects the segmentation accuracy of the 3D surface-rendered volumetric models derived from cone-beam CT, which to our knowledge has not been studied previously. Well-defined linear and angular measurements combined with volume comparisons demonstrated as an accurate tool to measure differences between 3D digital models were used as a method to illustrate the soft tissue effect [41–43]. Our results indicate that the soft tissue presence seems to affect the accuracy of the 3D hard tissue model of both the maxillo-facial complex and the mandible obtained from a cone-beam CT, however, below a generally accepted level of clinical significance of 1 mm [44]. It must nevertheless be noted that this level of accuracy may not meet the requirements of all applications, especially where higher precision is paramount [45, 46].

All segmentations, linear and angular measurements, and volume comparison procedures were performed repeatedly by one single operator. The repeatability of the segmentation process was found excellent with the differences between repeated segmentations below the set voxel size of 0.3 mm. The ICCs of the linear and angular measurements were classified as “excellent” which is in agreement with previous studies reporting excellent reliability of this method within and among different observers [43, 47]. The differences of the linear and angular measurements for the maxillo-facial complex were below 1 mm. In the mandible, larger differences were observed which could have possibly demonstrated a considerable effect of soft tissue on the inaccuracy of the models. However, carefully analyzing these results and taking into consideration the maceration and drying process involved in this study, such a conclusion might be found misleading. Previous studies showed an effect of drying on bone morphology particularly concerning a mandible. The posterior transversal dimension of the pig mandibles were affected up to an extent of 2.7 % [48]. This is in agreement with the present study, in which both the linear and angular measurements and the superimpositions showed differences for the mandibular transversal measurements. Although comparing the maxillo-facial complexes did not reveal such dimensional distortion as seen in the mandible, we could not presume that this region was not affected. In order to minimize any effect of the possible transversal deformation due to the maceration and drying process involved, the mandible and the maxillo-facial complex models were split in their midsagittal planes and the left and right parts were registered and compared separately. The characteristic measure of the inaccuracy of a tested model compared to the reference is the root-mean-square error or RMSE that serves as a measure of how far is the average error from 0, i.e., the distance difference, between the surfaces of the two models. Whereas the mean RMSE values of the maxillo-facial complex barely differ before and after the splitting, that of the mandible significantly decreased to less than 1 mm, which is generally considered as a clinical acceptable threshold [44]. Further, all mean differences between the surfaces were negative values. This shows a trend in which the dry skull models lie slightly within the cadaver models when the two models were optimally superimposed, indicating the dry skull models being smaller in general which could be caused by a change in humidity [39]. Although this is a consistent finding, the differences never exceeded the limits of the voxel size of 0.3 mm used in this study. These findings confirm the results from previous studies demonstrating that interpretation of research data, in which dry skulls were used, especially concerning the mandible, should be analyzed with the knowledge that significant changes may have taken place in the cranio-facial dimensions [48].

Special consideration was taken to control the possible affecting parameters, in order to focus on the effect of soft tissue presence in the accuracy of the 3D model, although this would partly limit the external validity of this study. All dry skulls were scanned using the same cone-beam CT unit, with identical scan settings and similar positioning of the skull in the field of view. Given the fact that the voxel size is another affecting parameter, adjusting the setting to a higher scan resolution could compensate the effect of soft tissue presence. Concerning this parameter, it has been indicated that the accuracy of the models appears to be connected to the voxel size [49], although the differences were small. However, others did not find increased accuracy of linear measurements on segmented surface models decreasing the voxel size from 0.4 to 0.25 mm [34]. In our study, the voxel size was fixed to 0.3 mm.

Head positioning is also considered as a possible affecting parameter. The accuracy of linear measurements using wires glued on the skull reported no clinically relevant effect [50]. Head positioning has been shown to be an affecting variable in the accuracy of the 3D cephalometric measurements based on 3D CBCT surface images and the reliability of linear measurements in some studies [50–52] and to be an insignificant factor for measurement accuracy in others [19]. Since the range of positioning deviations for error-free measurements are not yet known, we specifically aimed at achieving similar positioning using the laser reference lines of the unit to control the positioning as a parameter affecting the accuracy.

Two other parameters assumed to have a possible effect on the segmentation accuracy are the operator performing the segmentation process and the software utilized. The software package employed requires the operator to define individual hard tissue threshold values for each skull and cadaver. A previous study showed that a commercial software company produced more accurate surface models compared to an experienced 3D clinician [33]. This higher accuracy could be the result of a more experienced operator performing the segmentation or ascribed to the use of different tools and methods provided by different software packages. Both these affecting factors were well controlled in this study by utilizing one operator with a high intraobserver reliability performing segmentations on both the cadaver and dry skull and the use of one professional software package.

This study used a limited but unique sample of paired CBCT DICOM datasets from seven human cadaver heads and their respective dry skulls, contributing to a method in which the surface models with and without a natural soft tissue coverage could be compared. The effect of the scattering radiation due to the presence of soft tissues, resulting in deterioration of the signal-to-noise ratio and subsequently of the gray-level value differentiation in voxels, was well simulated. The method has a clear advantage over the use of artificial media, such as water balloons, to mimic the coverage of soft tissues [32, 53]. However, it has to be acknowledged that this method still contains a number of limitations. The preservation and storage of the cadavers was done in formaldehyde embalming fluid which could have increased the soft tissue thickness, altered the bone properties of the cadaver heads, and subsequently influenced the scattering radiation during the radiographic examination [54]. Therefore, in this study, the cadaver heads were not meant for representing the exact human anatomy of a living being. For the purpose of the present study, they were merely used as ex vivo equivalent of human heads to investigate the effect of soft tissue coverage.

Further research should focus on other affecting parameters resulting in a 3D surface-rendered model including the hardware, software, and operator-dependent parameters. By adjusting and improving these parameters, the effect of soft tissue presence should be minimized resulting in more accurate 3D surface-rendered models suitable for high-accuracy demanding applications.

## Conclusion

Considering the inherent limitations of any method involving preparation of dry skulls, it seems that the soft tissue presence does affect the segmentation accuracy of the 3D hard tissue model of both the maxillo-facial complex and the mandible obtained from a cone-beam CT, however, below a generally accepted level of clinical significance of 1 mm. As this level of accuracy may not meet the requirements of applications where high precision is paramount, further studies should investigate how to optimize the setting parameters to overcome the potential inaccuracy of CBCT-derived surface models related to the presence of soft tissue.
